# Peer-to-Peer Social Media Communication About Dietary Supplements Used for Weight Loss and Sports Performance Among Military Personnel: Pilot Content Analysis of 11 Years of Posts on Reddit

**DOI:** 10.2196/28957

**Published:** 2021-10-04

**Authors:** Kendall J Sharp, Julia A Vitagliano, Elissa R Weitzman, Susan Fitzgerald, Suzanne E Dahlberg, S Bryn Austin

**Affiliations:** 1 Department of Social and Behavioral Sciences Harvard T. H. Chan School of Public Health Boston, MA United States; 2 Division of Adolescent and Young Adult Medicine Boston Children's Hospital Boston, MA United States; 3 Department of Pediatrics Harvard Medical School Boston, MA United States

**Keywords:** dietary supplements, social media, Reddit, OPSS

## Abstract

**Background:**

Over 60% of military personnel in the United States currently use dietary supplements. Two types of dietary supplements, weight loss and sports performance (WLSP) supplements, are commonly used by military personnel despite the associated serious adverse effects such as dehydration and stroke.

**Objective:**

To understand peer-to-peer communication about WLSP supplements among military personnel, we conducted a pilot study using the social media website, Reddit.

**Methods:**

A total of 64 relevant posts and 243 comments from 2009 to 2019 were collected from 6 military subreddits. The posts were coded for year of posting, subreddit, and content consistent with the following themes: resources about supplement safety and regulation, discernability of supplement use through drug testing, serious adverse effects, brand names or identifiers, and reasons for supplement use.

**Results:**

A primary concern posted by personnel who used supplements was uncertainty about the supplements that were not detectable on a drug test. Supplements to improve workout performance were the most frequently used.

**Conclusions:**

Our pilot study suggests that military personnel may seek out peer advice about WLSP supplements on Reddit and spread misinformation about the safety and effectiveness of these products through this platform. Future directions for the monitoring of WLSP supplement use in military personnel are discussed.

## Introduction

The dietary supplement market in the United States is enormous and steadily growing, with a projected $56.7 billion in annual revenue by 2024 [1]. Supplements are widely available both online and in brick-and-mortar retail outlets across the country. Recent estimates suggest that approximately 52% of the adult population [2] and 60%-70% of military personnel [3] in the United States have used dietary supplements. A meta-analysis by Santos et al [4] found that approximately 10% of the adult population in the United States has used dietary supplements specifically for weight loss [4]. Many dietary supplements are marketed with claims that these suppress appetite, reduce fat absorption, increase the body’s ability to burn fat through increased metabolism, and decrease water weight [4]. Sports performance supplements are marketed with claims that these increase energy, improve sports or workout performance, and build muscle. These performance supplements are popular among military personnel, who have reported higher use of these types of supplements than civilians [5]. In a random sample of soldiers on active duty in the army, Lieberman et al [5] found that 25% of soldiers who reported dietary supplement use did so to increase their muscle strength and 17% did so to enhance their physical performance [5].

The Dietary Supplement Health and Education Act of 1994 allows the US Food and Drug Administration (FDA) to loosely regulate supplements, but the FDA cannot mandate rigorous testing and reporting of the efficacy, safety, or purity of dietary supplements before these are placed on the market [6]. These weak regulatory conditions have led to companies marketing dietary supplements with inaccurate efficacy claims and using undisclosed proprietary formulas without rigorous premarket testing [6].

Weight loss and sports performance (WLSP) supplements have been associated with a myriad of adverse health effects, including dehydration, electrolyte imbalance, digestive dysfunction, stroke, renal failure, and death [7-11]. A study of preworkout supplement use among adults in the United States found that over half of the participants who used these supplements reported adverse effects such as lightheadedness, rapid heart rate, and nausea [12]. Despite the large market and well-documented health risks, there is no national systematic surveillance to assess the prevalence of use or adverse effects of these products in the general population.

Military personnel are particularly vulnerable to using these products and adopting other dangerous weight control behaviors because of their rigorous training regimens and long work hours and the need to reach a certain weight to be eligible for military service [13,14]. Dietary supplement use has been associated with serious adverse effects, including death, in military personnel [15,16]. For instance, Eliason and colleagues [15] reported the cases of 2 soldiers who died after ingesting dietary supplements containing 1,3-dimethylamylamine, a powerful stimulant that is a derivative of amphetamine [15]. The use of supplements containing ephedrine, which can have similar life-threatening effects, has long concerned the air force and other branches of the military [17]. Ephedrine was routinely advertised to increase energy and weight loss, but its use in dietary supplements was prohibited by the FDA in 2004 [18]. To circumvent the FDA ban on dietary supplements containing ephedrine, some consumers create an ephedrine, caffeine, and aspirin (ECA) supplement mix, colloquially referred to as an ECA or EC (ephedrine and caffeine) stack, and often combine over-the-counter drugs and supplements or food sources as ingredients for this mix [19]. ECA stacks are commonly used as preworkout supplements, with the expectation that this mix will increase performance during workouts or reduce body fat—colloquially referred to as “cutting” fat—and lead to weight loss.

In 2012, the US Department of Defense (DoD) created the Operation Supplement Safety (OPSS) campaign to increase awareness of the dangers of dietary supplement use [20]. The OPSS website was created to educate military personnel about the risk of supplement use and to address questions that personnel may have in general on dietary supplements and on supplement safety and legality. The OPSS website also has a portal for voluntary reporting of adverse effects experienced after dietary supplement use. Despite the serious risk to service members and troop readiness and the concerning ad hoc adverse effects reported through the OPSS website, the DoD does not conduct routine systematic surveillance of the use of these dietary supplements or the resulting health problems in these personnel.

Social media platforms, such as Reddit, have been employed to understand the mental health needs and experiences of military personnel [21]. Because there is no routine systematic surveillance system for dietary supplement use in military personnel, social media platforms can help researchers better understand peer-to-peer communication about the use and patterns of use of these products. As of 2019, the social media site Reddit has 430 million active monthly users, and all Reddit data are publicly available and can be used for research [22]. Reddit allows users to follow and post on specific topics in communities called subreddits. Any user can create a new subreddit or post in existing subreddits. There are subreddits specifically for military personnel broken down by military branch, such as the air force or army. In these subreddits, users can post and comment in a thread format on a variety of topics. This thread format allows users to obtain opinions and viewpoints from any Reddit user.

In several studies, Reddit has been used to assess peer-to-peer communication on topics such as mental health [23], eating disorders [24], substance use [25,26], and vaping [27-29]. Coelho et al [21] conducted a study of military personnel subreddits on Reddit to assess veterans’ experiences with posttraumatic stress disorder. To our knowledge, no studies have examined discussions on supplement use by military personnel on Reddit. Therefore, we conducted a pilot study with the primary aims to assess whether military personnel subreddits have content on WLSP supplement use and to estimate intercoder agreement and a secondary aim to identify the main content of peer information on this topic. We anticipate the findings of this study will inform our understanding of the feasibility of a larger future investigation on the patterns of use of WLSP supplements among military personnel and peer-to-peer messaging around these supplements in service member spaces.

## Methods

Our pilot study used data from Reddit to examine peer-to-peer communication about WLSP supplements among military personnel. The Boston Children’s Hospital institutional review board determined this study to be exempt from the need to obtain informed consent given the nonidentifying nature of the data.

### Data Source and Platform

Reddit hosts the following 6 publicly available military personnel subreddits for the air force, army, military, navy, and coast guard: r/AirForce, r/army, r/Military, r/navy, r/uscg, and r/USMC. To identify all potentially relevant posts between January 1, 2009, and December 31, 2019, we used the following search terms: “supplements,” “dietary supplements,” “ECA stack,” “EC stack,” and “drug test.” A total of 570 potentially relevant posts were extracted for review to identify those related to WLSP supplements. Posts related to vitamins and other types of dietary supplements were excluded. A total of 64 relevant posts, to which users posted 243 relevant comments in response, were included in the analytic database for this study.

### Data Capture and Coding

Posts and comments were captured as PDF files and imported into QSR International’s qualitative software, NVivo 12, for coding [30]. Our study followed a data collection and reporting protocol similar to that used by Sowles and colleagues [26]. An inductive coding protocol created from commonly posted information in the Reddit posts and key terms from recent literature on WLSP dietary supplements were used to categorize posts and comments by year of posting (2009-2019) and subreddit. These posts and comments were then coded by two independent coders (KJS, JAV) for the following 6 key themes: the OPSS website, research on WLSP dietary supplements, discernability of supplement use through drug testing, brand names or identifiers, serious adverse effects, and reason for supplement use.

One of the primary aims of our pilot study was to evaluate the intercoder agreement for the Reddit posts. We assumed that feasibility would be achieved if the lower bound of the two-sided 95% exact binomial CI for the proportion of successfully coded subreddits exceeded 80%; this is equivalent to observing successful coding of at least 58/64 (91%) posts. Cohen κ [31] was used to evaluate the interrater reliability of the two ratings of the 6 key themes, and CIs for the κ coefficient were estimated using the method developed by Fleiss, Cohen, and Everitt [32]. Statistical significance was defined as a two-sided *P*<.05, and no adjustments were made for multiple comparisons. Cohen κ values in the range of 0.61-0.80 indicate substantial agreement, and values >0.80 indicate almost perfect agreement [33]. Cohen κ results indicated very strong agreement between the two coders’ evaluations, with all *P* values <.001 (Table 1).

The word frequency analysis tool in NVivo 12 was used to identify words that were frequently used in the collected Reddit posts and comments. The tool identifies exact matches for strings of 3 or more characters. Frequent words were analyzed if they appeared in the Reddit posts 3 or more times. Common words including “the,” “like,” “and,” and “but” were excluded from the results.

**Table 1:** Interrater agreement for coding of Reddit post themes appearing in military personnel subreddits from 2009 to 2019 (N=64 posts).

Themes	κ value (95% CI)	*P* value
Operation Supplement Safety	0.85 (0.56-1.0)	<.001
Other research cited^a^	0.74 (0.39-1.0)	<.001
Drug testing/positive test result	0.92 (0.75-1.0)	<.001
Products and ingredients	0.97 (0.91-1.0)	<.001
Reasons for use	0.97 (0.91-1.0)	<.001
Adverse effects	1.0^b^ (1.0-1.0)	N/A^c^

^a^Research from sources other than Operation Supplement Safety (eg, the US Food and Drug Administration website).

^b^Variance was estimated to be zero as a result of perfect agreement between coders.

^c^N/A: not applicable.

## Results

Peer-to-peer communication about WLSP supplements was seen in all 6 military personnel subreddits beginning in the year 2012, but no relevant posts were identified from 2009 to 2011 (Table 2). The largest number of relevant posts (23/64, 36%) was seen in the r/AirForce subreddit and in 2019 (16/64, 25%). A total of 64/64 (100%) posts in the military subreddits were successfully coded by both coders, which indicated the feasibility of our pilot study. The two-sided 95% exact binomial CI for this proportion is 94.4%-100%. Of the 64 total posts, 60 (94%) were posts asking for advice, and 4 (6%) were posts with general information.

Terms related to drug testing, such as “drug test,” “illegal,” “banned,” and “pop,” appeared frequently in posts and comments, suggesting that users were concerned with which WLSP supplements were legal and which might lead to a positive result on a drug test (Table 3). Other frequently used terms were those related to places from where supplements could be bought, such as “base,” “GNC,” and “online.” General Nutrition Center (GNC) stores and other stores on military bases were the most frequently mentioned places from where these products could be bought. Reddit users mentioned the OPSS website 23/307 (7.5%) times in the posts and comments, and they mentioned this site in a way that conveyed it to be a source for determining if a supplement was allowed for use by military personnel. Preworkout and bodybuilding were the most frequently mentioned reasons for purchasing and using these supplements.

As shown in Table 2, ECA or EC stacks were mentioned 27/307 (8.8%) times in the Reddit posts and comments. The most commonly misused drug used in place of ephedrine in ECA stacks was Bronkaid—an over-the-counter asthma medication easily available for purchase in most pharmacies in the United States—which was specifically mentioned 8/307 (2.6%) times in the posts and comments.

**Table 2:** Keywords in military personnel subreddit posts and comments by year (2009-2019) and subreddit.

Reddit post information		Frequency of words, n
**Keywords searched (N=307)**		
	Supplements/dietary supplements	232
	Drug test	84
	EC^a^/ECA^b^ stack	27
**Number of relevant posts per subreddit (N=64)**		
	r/AirForce	23
	r/army	13
	r/Military	11
	r/navy	8
	r/uscg	2
	r/USMC	7
**Number of relevant posts per year (N=64)**		
	2009	0
	2010	0
	2011	0
	2012	7
	2013	5
	2014	5
	2015	4
	2016	9
	2017	8
	2018	10
	2019	16

^a^EC: ephedrine and caffeine.

^b^ECA: ephedrine, caffeine, and aspirin supplement mix.

**Table 3:** Frequently used words representing key themes in military personnel subreddit posts (N=64) and comments (N=243) from 2009 to 2019.

Key theme			Frequency of words, n
**Operation Supplement Safety**			
	Link to Operation Supplement Safety website	23	
**Other research cited^a^**			
	Food and Drug Administration	30	
	Department of Defense	25	
**Drug testing/positive test result**			
	Banned	93	
	Illegal	30	
	Pop/popped/popping	53	
**Products and ingredients**			
	Animal Pak	20	
	Bronkaid	8	
	Caffeine	12	
	DMAA^b^	46	
	Ephedrine	32	
	Ephedra	7	
	Jack3d	17	
	Pseudoephedrine	4	
	Stack	16	
	Tea	3	
**Reasons for use**			
	Bodybuilding	12	
	Detox	3	
	Preworkout	60	
**Where to purchase**			
	Base (ie, military base)	22	
	GNC^c^	31	
	Online	14	
	Over the counter	12	

^a^Research from sources other than Operation Supplement Safety (eg, the US Food and Drug Administration website).

^b^DMAA: 1,3-dimethylamylamine.

^c^GNC: General Nutrition Center.

Table 4 presents exemplar quotes showcasing each of the 6 key themes. Similar to the word frequency results, the quotes illustrate peer-to-peer communication by Reddit users on topics such as the safety of preworkout supplements and the likelihood of a positive result on a drug test. Adverse effects of WLSP supplement use were mentioned, including elevated heart rate and death. Many posts included misinformation about these supplements; one user stated, “if you buy [supplements] at GNC, you’re good to go,” with respect to passing a drug test, though this view was not consistent across users. Some users posted that they had failed drug tests after using supplements purchased at GNC or other stores on their base.

Reddit users asked their subreddit peers about ECA stacks, where to purchase the ingredients, and if the use of these supplements would lead to a positive result on a drug test. Several users shared similar reasons for using ECA stacks, as expressed by one user who wanted to “cut some fat and [get] below [my] max weight.” The OPSS website and other research sites, such as the FDA website, were mentioned as resources by some Reddit users.

**Table 4:** Exemplar quotes related to weight loss and sports performance dietary supplements for each key theme appearing in military personnel subreddits from 2009 to 2019.

Theme and subreddit			Year of posting		Quote		Coded themes
**Drug testing/positive test result**							
	r/army	2012		“Just curious if supplements such as preworkouts or proteins are allowed in basic?”		Regulation of supplements Preworkout	
	r/army	2012		“…the Army (and pro-sports) has banned any preworkout with 1,3 Dimethylamylamine [DMAA] in them (such as Jack3d and Oxyelite Pro).”		Regulation of supplements DMAA^a^ Jack3d OxyElite Pro	
**Operation Supplement Safety**							
	r/navy	2019		“https://www.opss.org/dietary-supplement-ingredients-prohibited-department-defense Looks like it’s listed under the ‘unapproved drugs’ section.”		Operation Supplement Safety Regulation of supplements	
**Other research cited^b^**							
	r/army	2019		“If you are ordering stuff from sketchy suppliers on the internet, who knows what’s in it. https://www.fda.gov /ForConsumers/ConsumerUpdates/ucm 173739.htm”		Other research Regulation of supplements	
**Products mentioned**							
	r/AirForce	2015		“Are we allowed to take EC stack? I’ve heard anecdotes of people saying you can take it and there’s no problem, but just to be clear, ephedrine can cause false positives for methamphetamine. You have to buy Bronkaid or Primatene at a pharmacy in order to use the EC stack because ephedrine isn’t sold as a standalone product.”		Positive test result EC^c^ stack Ephedrine Bronkaid	
	r/army	2018		“I am thinking of starting an E/C stack taking Bronkaid (OTC) and Caffeine pills in order to cut some fat and get below my max weight. I wanted to just ensure that this is ok for me to do, and that it wouldn't get me in any sort of trouble. I appreciate any and all advice, thanks!”		Regulation of supplements Weight loss Bronkaid EC stack	
	r/navy	2019		“E/C stack is about the only stack that's not a waste of money”		EC stack	
**Reason for supplement use**							
	r/USMC	2018		“Want to take a preworkout with DMHA in it. DMAA is on the banned substance list, but DMHA is not. If I pop a false positive on a drug test, will I get charged? Or will they do further tests and determine it was a legal stimulant in a pre-workout?”		Regulation of supplements Preworkout DMAA DMHA^d^	
**Adverse effects**							
	r/AirForce	2019		“PSA for all you PT/Mock test takers. Do not, under any circumstances, take multiple energy pills you bought at the gas station before you test. Never thought it would be a problem, but I was testing one of our officers this morning. He completed 1 lap and proceeded to vomit. His heart rate was over 210 and I was starting to worry that we would have to call an ambulance.”		Vomiting Elevated heart rate Energy supplements	
	r/army	2019		“jack3d apparently did [make you test positive on a drug test] back in like 2009-2010. I used to use it lmao but never popped hot or anything. The military banned it and the product changed to remove some sort of amphetamine it had. It also killed a [couple] dudes in the Army and at USMA though from heart attacks so it could also have been that.”		Regulation of supplements Drug testing Jack3d Heart attack	

^a^DMAA: 1,3-dimethylamylamine.

^b^Research from sources other than OPSS (eg, the US Food and Drug Administration website).

^c^EC: ephedrine and caffeine.

^d^DMHA: 1,5-dimethylhexylamine.

## Discussion

To our knowledge, our pilot study is the first to examine peer-to-peer communication about WLSP supplements on a social media platform and document messaging around these products within all 6 publicly available military personnel subreddits from 2009 to 2019. Our results suggest that military personnel are interested in using these supplements regardless of existing DoD regulations and the potentially serious adverse effects.

The DoD has prohibited the use of some of these types of supplements because of the associated serious adverse effects [15], but the supplements that were banned for use were not clear to all Reddit users. A total of 60/64 (94%) posts were questions about supplement types, drug testing, or supplement use, while only 7/243 (2.9%) comments referenced the OPSS website. Thus, more education on this important resource is needed for service members [20].

The results of this study suggest that there is interest in using muscle-building and preworkout supplements among military personnel despite DoD prohibitions and the risk associated with the use of some types of supplements. ECA stacks were frequently mentioned in these subreddits and seemed to be favored by Reddit users over preformulated performance and preworkout supplements because of the perceived effects of the ECA ingredients on the body. Reddit users posted simple instructions for their peers explaining how users can make their own ECA stack from easily obtained ingredients. Many users suggested that the supplements had the effect of “cutting” fat, which would help them reach the weight required for service. Our results showed that the r/AirForce subreddit had the highest number of posts, but it is unclear if WLSP supplement use is higher in this branch than in the other branches of the military.

A limitation of this pilot study is that Reddit does not provide any demographic information on their users. Therefore, we could not determine the location, age, or gender of these users. There may be important differences in these demographic characteristics among military personnel who use these products. In addition, the subreddits included in our study were publicly accessible; although these communities are described as affinity groups for military personnel in different branches, it is possible that nonmilitary Reddit users join and post on these subreddits. Another limitation is that our research team could not access certain subreddits. For example, r/Marines is a private subreddit and a user has to be invited and approved by a moderator to access the subreddit. We do not know if WLSP supplements are discussed in these subreddits and—if they are—whether these conversations are similar to those in the publicly available subreddits.

Our investigation was restricted to Reddit to evaluate the feasibility of studying peer-to-peer communication about WLSP supplements among military personnel on a social media platform. Our findings suggest that such a study is not only feasible but can also provide novel insight into peer-to-peer communication about beliefs, misconceptions, concerns, and behaviors vis-à-vis WLSP supplements. Future research should focus on other social media platforms to examine similar communication among military personnel and other populations.

With the current use of dietary supplements at over 60% for military personnel [3], future studies should investigate WLSP supplement use patterns by branch of service and sources of supplements purchased, frequency of failed drug tests attributable to WLSP supplement use, chemical composition and safety of commonly used WLSP supplements, and adverse effects experienced by military personnel using WLSP supplements. The danger these products pose to the health and safety of military personnel is becoming increasingly clear even through current ad hoc channels for reporting adverse effects, which raises serious concerns about the negative impact of WLSP supplement use on the overall military readiness of the nation’s armed forces. Through the OPSS program, the DoD may consider further steps to address the use of these types of dietary supplements by military personnel. For instance, the DoD could disseminate more information for military personnel and military medical providers on the dangers of WLSP supplement use. DoD leadership could consider implementing routine, systematic surveillance to track the use of WLSP supplements within the military. Finally, the DoD could consider more stringent restrictions on the sale of these products on military bases.

## Abbreviations

DoD: Department of Defense
ECA: ephedrine, caffeine, and aspirin
EC: ephedrine and aspirin
FDA: Food and Drug Administration
GNC: General Nutrition Center
OPSS: Operation Supplement Safety
WLSP: weight loss and sports performance
